# Relation between Headache and Mastication Muscle Tone in Adolescents

**DOI:** 10.1155/2018/7381973

**Published:** 2018-09-23

**Authors:** Ewa Wozniak, Jolanta E. Loster, Aneta Wieczorek

**Affiliations:** Department of Dental Prosthetics, Institute of Dentistry at Jagiellonian University, Krakow, Poland

## Abstract

Headache is a common problem in the population, which decreases the quality of life and makes everyday functioning difficult. It often coexists with typical symptoms of temporomandibular disorders. The objective of the study was to clarify whether there is a relationship between the presence of headache in young volunteers and the mastication muscle tone. *Material and Method*. Volunteers aged 18 years who underwent general dental examination, clinical evaluation, and examination using the dual-axis diagnostic system Research Diagnostic Criteria for Temporomandibular Disorders (RDC/TMD) form in the Polish language version participated in the study. On the basis of the examination results, these individuals were divided into three groups according to RDC results. A group of healthy individuals (axis I value 0), a group of sick individuals (axis I diagnosis Ia, IIa, or IIIa), and a group of individuals suffering from depression (axis II values 1–3) were singled out. Then, volunteers were divided into two groups: group I reporting headache and group II without any pain. In each of these individuals, the contractile activity of the masseter muscles and anterior temporal muscles was recorded bilaterally as the surface electromyographic activity (sEMG) at rest, during swallowing, and maximum clenching. The activity index ranging between +100 (activity of the masseter muscles only) and −100 (activity of the anterior temporal muscles only) was used to indicate the predominance of activity of the study muscles. *Results*. The statistical analysis of data showed that there was a significant relationship between the presence of headache and a change in the mastication muscle tone, expressed as the predominant activity of the temporal muscles, only in the group of sick individuals during maximum clenching. *Summary*. The diversity of sEMG results implies that a change in the mastication muscle tone is not a direct consequence of headache, but it has to be modified by other factors.

## 1. Introduction

Headache is a common condition which compromises everyday functioning and decreases the quality of life not only in adults. The results of the systematic review covering epidemiological studies on the prevalence of headache among children and adolescents from 32 countries have shown that the problem affects 54.4% of the population on average [1]. The causes of headache vary due to the great number and complexity of anatomical structures, various pathophysiological mechanisms, and effects of psychogenic factors. Temporomandibular disorders (TMD) are among conditions which may manifest with headache. This term covers a broad range of clinical problems that are related to the masticatory muscles, temporomandibular joints, and adjacent tissues [2]. Functional disorders are the most common orofacial pain syndrome due to which patients seek medical help [3]. According to the studies by Solberg et al., 76% of study subjects aged 18–25 years reported signs associated with TMD, and at least one typical sign of TMD was found in 26% [4]. In the past, the diagnosis of temporomandibular disorders was problematic, but today clinicians have diagnostic tools at their disposal that make it much easier to establish the diagnosis and than to apply appropriate treatment strategies. The standardized classification of TMD has become possible owing to the development of research diagnostic criteria for TMD [5]. This diagnostic system takes into account the biopsychological etiology of the analysed problem and comprises two axes: axis I is a set of procedures for clinical evaluation of a patient that lead to a physical diagnosis and axis II uses personal questionnaires with which, through the patient self-evaluation, the psychological dimension of pain, resultant disability, presence of depression, and nonspecific somatic symptoms can be determined. The results of scientific research show that typical symptoms of TMD-pain in the area of the temporomandibular joints and/or muscles of mastication, limited mandibular range of motion, and acoustic symptoms are more common in patients who suffer from primary headache, such as migraine or tension headache, compared to the healthy population [6, 7]. At the same time, the inverse trend has been observed, i.e., an increase in the prevalence of primary headache (in particular migraine) in patients with diagnosed TMD [8]. Moreover, it has been demonstrated that the coexistence of these two conditions exacerbates the typical symptoms of each of them, leading to a higher degree of disability [6]. Therefore, a conclusion can be drawn that procedures appropriate for the treatment of both headache and TMD should be included in the therapeutic management. One of TMD treatment strategies is to apply the therapy using occlusal splints in patients. There are scientific reports which confirm the efficacy of using occlusal splints not only for the elimination of mastication muscle pain and acoustic symptoms in the temporomandibular joints, but also for the treatment of migraine pain [9]. The results of studies involving electromyographic measurements of the mastication muscles indicate that there are effects such as a reduction in the electrical activity of the mastication muscles and equalization of the muscle activity on the left and right sides after the implementation of stabilization splint therapy [10]. Correlations between primary headache and TMD that are noticeable in the symptomatology and effects of similar treatment strategies suggest that there may be a relationship between headache and mastication muscle tone. The objective of the present study was to clarify whether there is a relationship between headache in young volunteers and the mastication muscle tone in the group of healthy individuals, group of individuals suffering from TMD, and group of individuals with diagnosed depression. The initial hypothesis assumed that the predominant activity of anterior temporal muscles would be observed in individuals with headache compared to individuals without any pain.

## 2. Material and Methods

Individuals aged 18 years were enrolled in the study from among volunteers taking part in the research project. None of these individuals had earlier contact with any of the study doctors or clinical procedures forming an integral part of the research project. Informed consent forms for participation in the project were signed by all subjects, and the study program was approved by the Bioethics Committee of the Jagiellonian University (KBET//89B/2009). The study was conducted in accordance with the Good Clinical Practice guide, Declaration of Helsinki.

The study inclusion criteria included full dental arches. The exclusion criteria included: malocclusions, carious destruction of dental tissues, permanent prosthetic restorations, therapy with botulinum toxin, mental illnesses, and pregnancy. Clinical evaluation was performed in each of the subjects by the same dental practitioner using the RDC/TMD dual-axis diagnostic system. A Polish version of the personal questionnaire forming a part of the RDC/TMD diagnostic algorithm was completed by the project participants themselves [12]. Based on the evaluation results and data from questionnaires, the subjects were assigned to one of 3 groups: group I of healthy individuals (RDC/TMD system axis I value 0), group II of sick individuals (axis I diagnosis Ia, IIa, or IIIa), and group III of individuals with depression or nonspecific somatic symptoms (axis II values 1–3). Each of the subjects was asked to assess the presence and intensity of headache on the day of evaluation using a 4 point scale: 0, no headache; 1, mild headache; 2, moderate headache; and 3, severe headache. All subjects underwent surface electromyography (sEMG) examination using the eight-channel BioEMG III BioPAK Measurement System Electromyograph (BioResearch, Inc., Milwaukee, WI, USA). Measurements of tone of the left and right masseter muscles (LMM, RMM) and the anterior temporal muscle on the left and right sides (LTA, RTA) were performed [13–16]. The first measurement involved the scenario with a position at rest, the second one was performed during maximum clenching, and the third one was taken during swallowing. For each of the scenarios, the activity index (AcI) was calculated, which was to indicate the predominant activity of the masseter muscle or the anterior temporal muscle. The activity index was calculated using the equation introduced by Naeije [17] in accordance with the following formula: AcI = (RMS_masseter_–RMS_temporal_)/(RMS_masseter_ + RMS_temporal_) × 100, where RMS is the root mean square. The activity index ranged between +100 (activity of the masseter muscles only) and −100 (activity of the anterior temporal muscles only). The value 0 meant that that there was a balance between the activity of both groups of muscles. The analysis of data consisted of the following steps:

1. Test for normality of distribution
2. Evaluation of the impact of presence of headache on the activity index of the mastication muscles in groups I, II, and III at rest
3. Evaluation of the impact of presence of headache on the activity index of the mastication muscles in groups I, II, and III during maximum clenching
4. Evaluation of the impact of presence of headache on the activity index of the mastication muscles in groups I, II, and III during swallowing

Statistical calculations were performed using the R 3.1.2 statistical package. The results with the *p* value < 0.05 were considered to be statistically significant.

## 3. Results

In total, 106 individuals (27 men and 79 women) aged 18 years were enrolled in the study. There were 74 subjects in the first group, 32 subjects in the second group, and the third group included 47 individuals.

**Figure 1:**
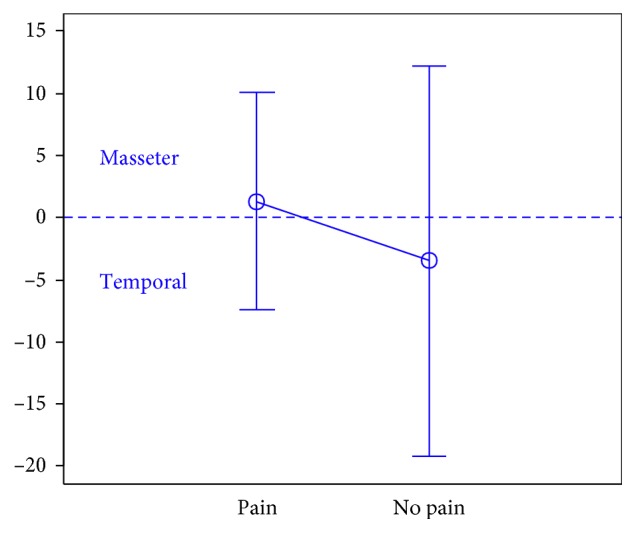
Activity index at rest in group of healthy individuals.

**Figure 2:**
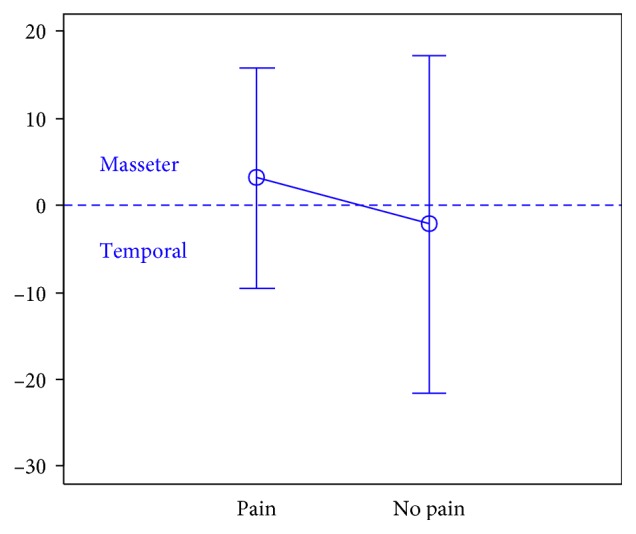
Activity index at rest in group of TMD individuals.

**Figure 3:**
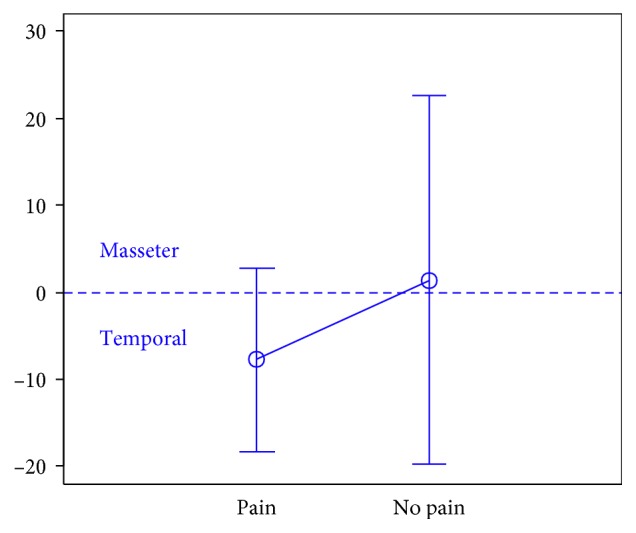
Activity index at rest in group of individuals with depression.

**Figure 4:**
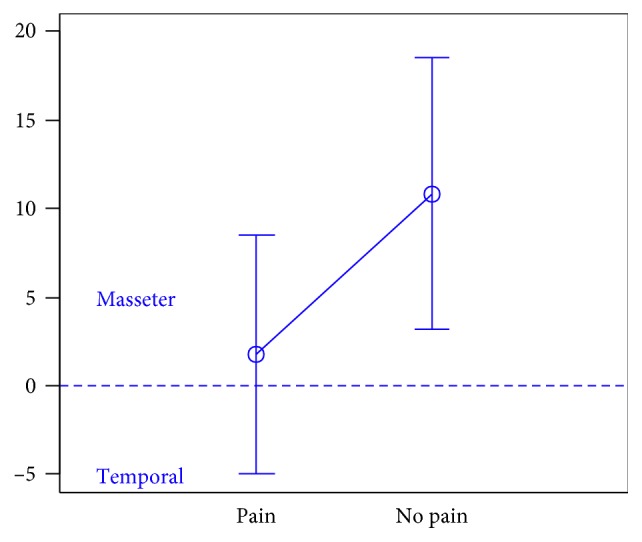
Activity index during maximum clenching in group of healthy individuals.

**Figure 5:**
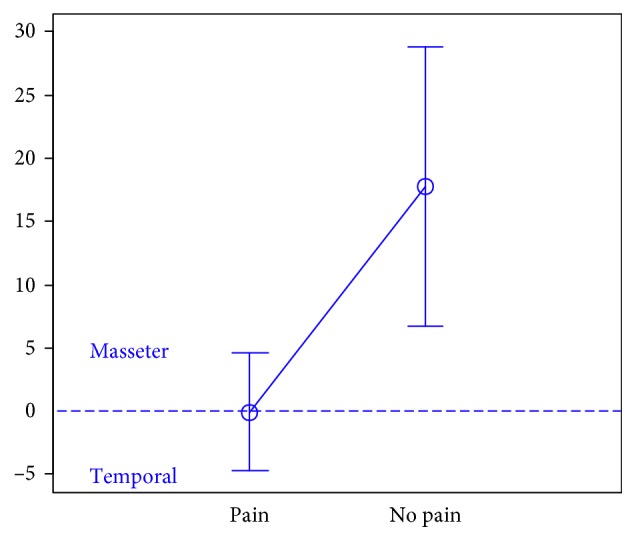
Activity index during maximum clenching in group of TMD individuals.

**Figure 6:**
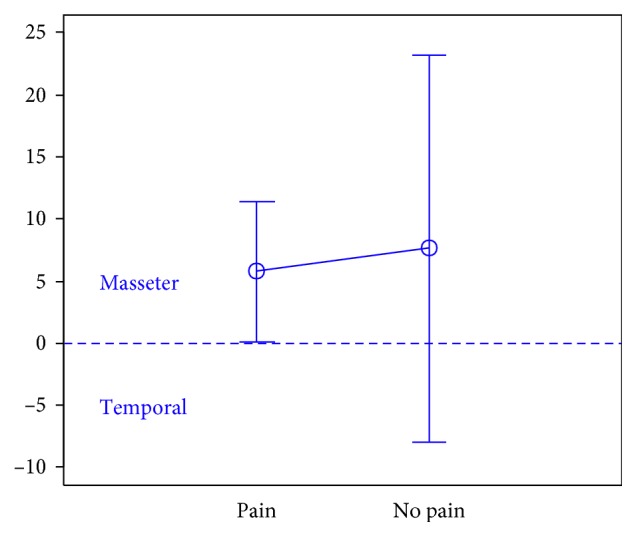
Activity index during maximum clenching in group of individuals with depression.

**Figure 7:**
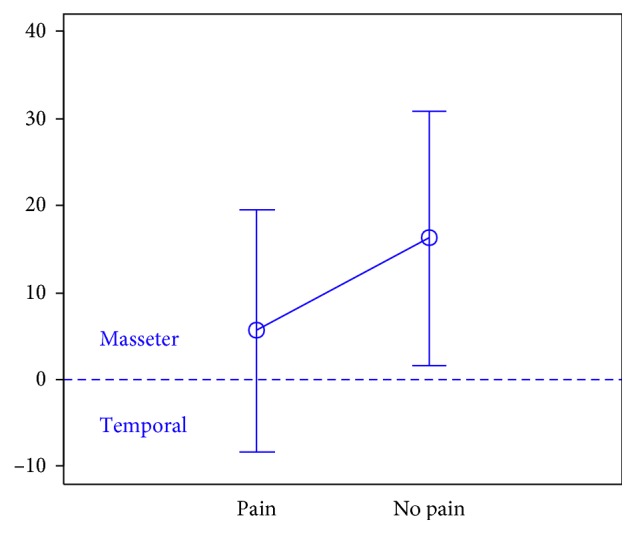
Activity index during swallowing in group of healthy individuals.

**Figure 8:**
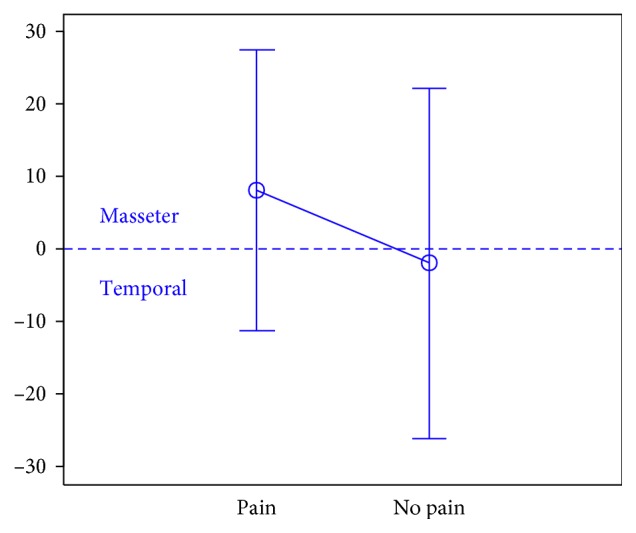
Activity index during swallowing in group of TMD individuals.

**Figure 9:**
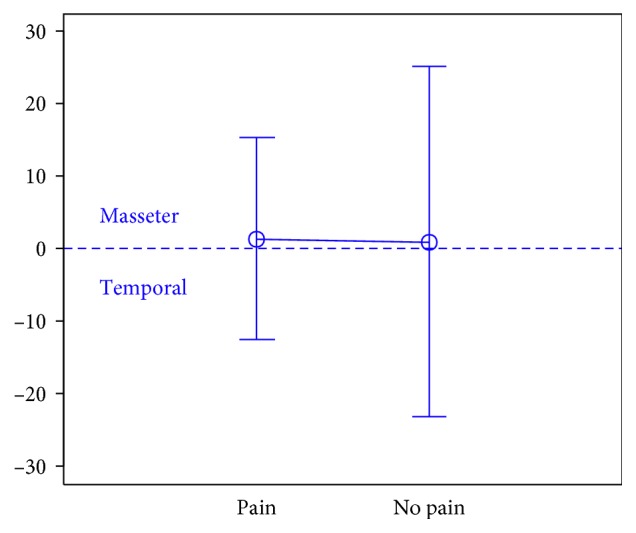
Activity index during swallowing in group of individuals with depression.

1. The activity indices of the mastication muscles were normally distributed across all groups under analysis (the *p* value of the Shapiro–Wilk test above 0.05), therefore the analysis was performed using the parametric Student *t-*test.
2. At rest, the differences in the activity index of the mastication muscles between individuals with headache and those without pain were not statistically significant in group I (healthy individuals), group II (sick individuals), and group III (individuals with depression) (group I: *p*=0.589; group II: *p*=0.641; group III: *p*=0.404) (Figures 1–3). Although there was no statistical significance, the activity index in group III indicated the predominance of the anterior temporal muscles in individuals with headache (AcI = −7.78), while in groups I and II, masseter muscles showed greater activity, if headache was present.
3. During maximum clenching, differences in the activity index of the mastication muscles between individuals with headache and those without headache in the group I were statistically insignificant (*p*=0.088); however, the activity index value was lower in individuals with headache compared to individuals without pain, which indicated a reduction in predominant activity of the masseter muscles. In the group II, the relationship between the presence of headache and a change in the activity index of the mastication muscles was statistically significant (*p*=0.003) (Figures 4–6). The mean AcI in sick individuals with headache was nearly 0 (AcI = −0.07), which indicated a balance between the activity of the masseter muscles and anterior temporal muscles, while AcI in sick individuals without pain was definitely positive (AcI = 17.73), which indicated the predominant activity of the masseter muscles. Differences in the activity index of the mastication muscles between individuals with headache and those without headache in the group III were not statistically significant (*p*=0.782); however, a tendency towards greater activity of the temporal muscles in individuals with pain was noticeable.
4. During swallowing, differences in the activity index of the mastication muscles between individuals with headache and those without headache were not statistically significant across all study groups (group I: *p*=0.309; group II: *p*=0.523; group III: *p*=0.972) (Figures 7–9). However, the increased activity of the temporal muscles was noticeable in healthy individuals in the case of presence of headache.

## 4. Discussion

The initial hypothesis of the present study was based on reports by Rodrigues-Bigaton et al. [11] who demonstrated that there was a statistically significant difference in the activity index of the mastication muscles during resting mandibular position in individuals with TMD compared to the healthy control group. In the study conducted by Rodrigues-Bigaton, the difference in the activity index was expressed as the dominance of the temporal muscles in individuals with TMD, so the relationship between the presence of TMD, of which mastication muscle pain is a component, and a change in the mastication muscle tone was demonstrated. The objective of the present study was to clarify whether the analogous relationship between the presence of headache and mastication muscle tone exists. Therefore, the initial hypothesis assumed that there would be a change in the mastication muscle tone expressed as the predominant activity of the temporal muscles in individuals with headache. The initial hypothesis was confirmed only in the group of sick individuals during maximum clenching. The results obtained by Rodrigues-Bigaton are in contrast to this observation. He found no statistically significant differences in the activity index of the mastication muscles between individuals with TMD and the control group in the situation of isometric contraction of the muscles of mastication. Although there was no statistical significance in the present study, tendencies in conformity with the initial assumption were noticeable in groups of individuals with depression in the position at rest and during maximum clenching and healthy individuals during maximum clenching and swallowing. Apart from studies conducted by Rodrigues-Bigaton, the issues related to the impact of mastication muscle pain on changes in the tone of these muscles during different functional activities were addressed by a number of other authors. There are scientific reports which found that there was no significant difference in the sEMG readings from the temporal muscles at rest between individuals with mastication muscle pain and the control group [18]. On the other hand, numerous studies on animals demonstrated that experimentally induced painful stimuli resulted in a short-term increase in the mastication muscle tone recorded in sEMG [19, 20]. The results of studies involving patients with TMD, who reported headache, also confirmed higher sEMG activity of the mandibular elevator muscles at rest in these patients [21]. However, it was observed that there were significant differences in the time of onset and duration of changes recorded in sEMG in individual subjects with pain. Some authors conclude that time parameters, such as onset and duration of changes in sEMG, may be more reliable in the identification of muscle activity patterns that are changed by pain than the amplitude itself of these changes [22]. The diversity of obtained results has provoked the development of contradictory hypotheses to clarify whether an increase in the sEMG reading for the mandible adductor muscles is caused by pain or may be a primary increase in the activity recorded in sEMG is a component of the feedback mechanism which maintains chronic muscle pain. The vicious cycle theory has been developed which assumes that a pain-initiating factor, e.g., abnormality in structure, posture, movement, or stress, leads spontaneously to muscular hyperactivity. It results in increased muscle tone, spasm and fatigue, and finally dysfunction which maintains pain [23]. A weak point of the mentioned theory is that the pathophysiological mechanism linking muscle pain and muscle tone has not been fully explained yet. It has been claimed that pain results in neuroplastic changes in the central nervous system, leading to increased motor neuron activity at supraspinal and segmental levels, of which the effect is slightly increased muscular activity [24]. It was also found that in patients reporting bilateral headache, the EMG readings from the mastication muscles were higher than these recorded in patients with unilateral pain [21]. It suggests that the modulation of muscular activity is not only a direct consequence of peripheral nociceptive mechanism and indicates a response involving the central nervous system as well. However, in the majority of studies, an increase in the EMG activity recorded when pain was present turned out to be so small (of about several *µ*V) that its role in the development of the claimed muscular hyperactivity and maintaining painful impulsation is doubtful; therefore, it seems to have no clinical relevance [23]. In the presented study a slight correlation between headache and a change in tone recorded superficially from the temporal and masseter muscles was confirmed. Some scientific reports assume the possibility that the examined muscles may show normal or slightly changed superficial EMG activity in pain syndromes, but certain specific, small areas within the muscles, so called trigger points, are distinguished by a higher EMG reading. Therefore, it is suspected that continuous activity of several motor units, which persists over a long period of time, may be sufficient for the stimulation of nociceptors [24], while the sEMG reading from the entire muscle remains unchanged. The pain adaptation model is the theory that explains the observed relationships better than the vicious cycle theory. It assumes that pain causes decreased EMG activity in the agonist muscles and results in reduced range of motion and its longer duration [23]. These changes are aimed at preventing further damage to the sensorimotor system. The present study results are in conformity with the mentioned theory: a significant change in the muscle tone was observed in individuals with diagnosed TMD who reported the presence of headache compared to individuals without any pain during maximum clenching of the teeth, i.e., during active contraction. Similar results were obtained by Li et al. [25] who investigated whether unilateral pain from TMD was associated with the occlusion contacts and EMG activity of the mastication muscles. They demonstrated that in individuals with TMD (with pain) the muscle tone measured using sEMG in both masseter muscles and the temporal muscle on the pain side was lower compared to the healthy control group during maximum clenching at maximum intercuspation. The newest model describing the relation between pain and muscle activity, i.e., the integrated pain adaptation model [23], assumes that pain results in a new, optimized recruitment strategy of motor units to minimize pain and maintain homeostasis. This model represents the individual response of the motor system to the multidimensional nature of pain: not only the biological dimension of pain but also psychosocial components of the pain experience are taken into account. Thus, it emphasizes the individual reaction to pain, i.e., factors such as the presence of depression, previous pain experience, social context, and genetic factors that will affect a change in the muscle activity in a unique way. The performed study did not demonstrate that the presence of headache in individuals with depression or psychosomatic symptoms had effect on the mastication muscle tone.

## 5. Conclusions

Headache has impact on a change in the mastication muscle tone in individuals with diagnosed TMD during maximum clench. The diversity of sEMG results implies that a change in the mastication muscle tone is not a direct consequence of headache, but it has to be modified by other factors.

## Data Availability

The data used to support the findings of this study are available from the corresponding author upon request.
